# Association between pelvic inflammatory disease and ovarian cancer: A bidirectional Mendelian randomization study

**DOI:** 10.1097/MD.0000000000049820

**Published:** 2026-07-24

**Authors:** Kaiyun Qin, Yan Jiang, Xingshuang Gao, Weilan Liu, Wenfei Wu, Junmei Zhang, Yuan Zhang, Zhaoping Chu

**Affiliations:** a Department of Gynecology, Hebei General Hospital, Shijiazhuang, Hebei, China; b Department of Obstetrics, Hebei General Hospital, Shijiazhuang, Hebei, China; c Physical Examination Center, Hebei General Hospital, Shijiazhuang, Hebei, China.

**Keywords:** factor analysis, Mendelian randomized, ovarian cancer, pelvic inflammatory disease

## Abstract

Some observational studies have suggested an association between pelvic inflammatory disease (PID) and ovarian cancer (OC), but the causality between PID and OC remains unclear. To investigate the causal association between PID and 7 OCs, we performed two-sample bidirectional Mendelian randomization (MR) analyses using genetic variants associated with PID. Pooled data on OC were obtained from the Ovarian Cancer Association Consortium. Effect estimates were obtained by inverse-variance weighting, the weighted median method, MR-Egger regression, and MR pleiotropy residual sum and outlier (MR-PRESSO) tests. The results of the inverse-variance weighting analysis showed that PID incidence risks were not significantly associated with OC, mucinous OC, low malignant potential OC, low-grade serous OC, high-grade serous OC, endometrioid OC, or clear cell OC. In this study, the causalities between PID and 7 OCs were comprehensively assessed, but the identified genetic evidence suggested no possible causalities between PID and 7 OCs in European populations.

## 1. Introduction

Cancer stands as the most prevalent cause of death in most regions worldwide.^[1]^ It remains a primary obstacle to achieving the desired life expectancy across many nations.^[2]^ Ovarian cancer (OC) has the highest fatality rates and poorest prognosis among malignant gynecological tumors.^[3]^ The heightened mortality associated with OC stems from challenges in early detection of lesions and a lack of effective late-stage treatments, earning it the moniker of a “silent killer.” Ovarian tumors are primarily classified histologically as epithelial, germ cell, stromal, or metastatic, with 85% to 90% falling under the category of epithelial ovarian tumors.^[4,5]^ Generally, tumors originate from 1 of the 3 cell types: epithelial cells, germ cells, or stromal cells, with those derived from epithelial cells dominating.^[6]^

The exact pathogenesis of OC remains unclear. In addition to recognized risk factors such as nulliparity, infertility, early menarche, delayed menopause, and a family history of OC, the use of talcum powder, endometriosis, and ovulation-inducing therapies have also been identified as risk factors for malignant ovarian tumors.^[7]^ These factors collectively involve or exacerbate the occurrence of local pelvic inflammatory responses.^[8]^ Recently, attention has been drawn to potential factors that might induce ovarian tumors, including pathogenic infections, endometriosis, and ovulation-inducing therapies, all of which can lead to or intensify pelvic inflammatory disease (PID).^[9]^ PID encompasses a group of diseases in the female reproductive tract caused by infections, including endometritis, salpingitis, tubo-ovarian abscess, and pelvic peritonitis.^[10]^ A study in Shanghai in 1989 first reported a risk association between PID and OC, though it was statistically insignificant.^[11]^ Subsequent research by Booth et al found that PID could increase the risk of OC, especially in cases of recurrent PID and at younger ages.^[12]^ Merritt et al^[13]^ indicated in a 2008 case-control study that chronic inflammation does not impact the development of OC. However, in 2011, Lin et al discovered a 1.9-fold increase in the development of epithelial OC among women exhibiting PID symptoms.^[14]^ Stewart et al’s article in 2018 revealed an increased risk of high-grade serous OC in women with pelvic inflammation.^[15]^ A meta-analysis conducted by Piao et al^[16]^ found an increased association between PID and OC risk (hazard ratio 1.18, 95% confidence interval [CI]: 1.13–1.22). These findings, however, remain inconsistent, with some studies suggesting a positive association and others finding no significant relationship, highlighting a persistent uncertainty in understanding whether PID truly plays a causal role in OC development. A major reason for these discrepancies is that conventional observational studies are inherently susceptible to residual confounding (sexual behavior, reproductive factors, and socioeconomic status) and reverse causality – where subclinical OC may itself influence the occurrence or diagnosis of PID. Such limitations make it difficult to disentangle correlation from true causation. Therefore, a methodological approach capable of minimizing confounding and establishing directionality is urgently needed to clarify the relationship between PID and OC.

Mendelian randomization (MR) is a statistical technique applied to epidemiology and genetics, with the objective of determining causalities between risk elements and outcomes.^[17]^ MR is based on the principles of Mendelian inheritance laws, which describe how genetic variants are assigned at random in the course of meiosis.^[18]^ This approach uses instrumental variables (IVs), particularly single nucleotide polymorphisms (SNPs) that are linked to the risk factor of interest (GERD) genetic variants, to detect whether the selected risk factors have an obvious causal effect on the outcomes of interest or not.^[19]^ Because genetic variants are fixed at conception and not influenced by lifestyle or disease status, MR can effectively reduce confounding and eliminate reverse causation, thereby providing stronger causal inference than traditional observational designs. Without randomized controlled trials, MR studies present an alternative tactic for causal inference.^[20]^ Thus, MR has superiority over conventional observational research in that it lessens the risk of confounding and reverse causality, making it an advantageous tool for detecting causality in epidemiological research.^[21]^ To date, no MR analysis has been conducted to evaluate the causal relationship between PID and OC or its histological subtypes. This represents a critical research gap, given the ongoing debate in epidemiological literature and the potential clinical implications of clarifying whether PID is a true causal risk factor. Therefore, this research aimed to establish a causality between PID and 7 OCs (OC: ovarian cancer; MOC: mucinous ovarian cancer; LMPOC: low malignant potential ovarian cancer; LGSOC: low-grade serous ovarian cancer; HGSOC: high-grade serous ovarian cancer; EndoOC: endometrioid ovarian cancer; CCOC: clear cell ovarian cancer) with a two-sample MR approach.

## 2. Materials and methods

### 2.1. Source of data

**Figure 1.**
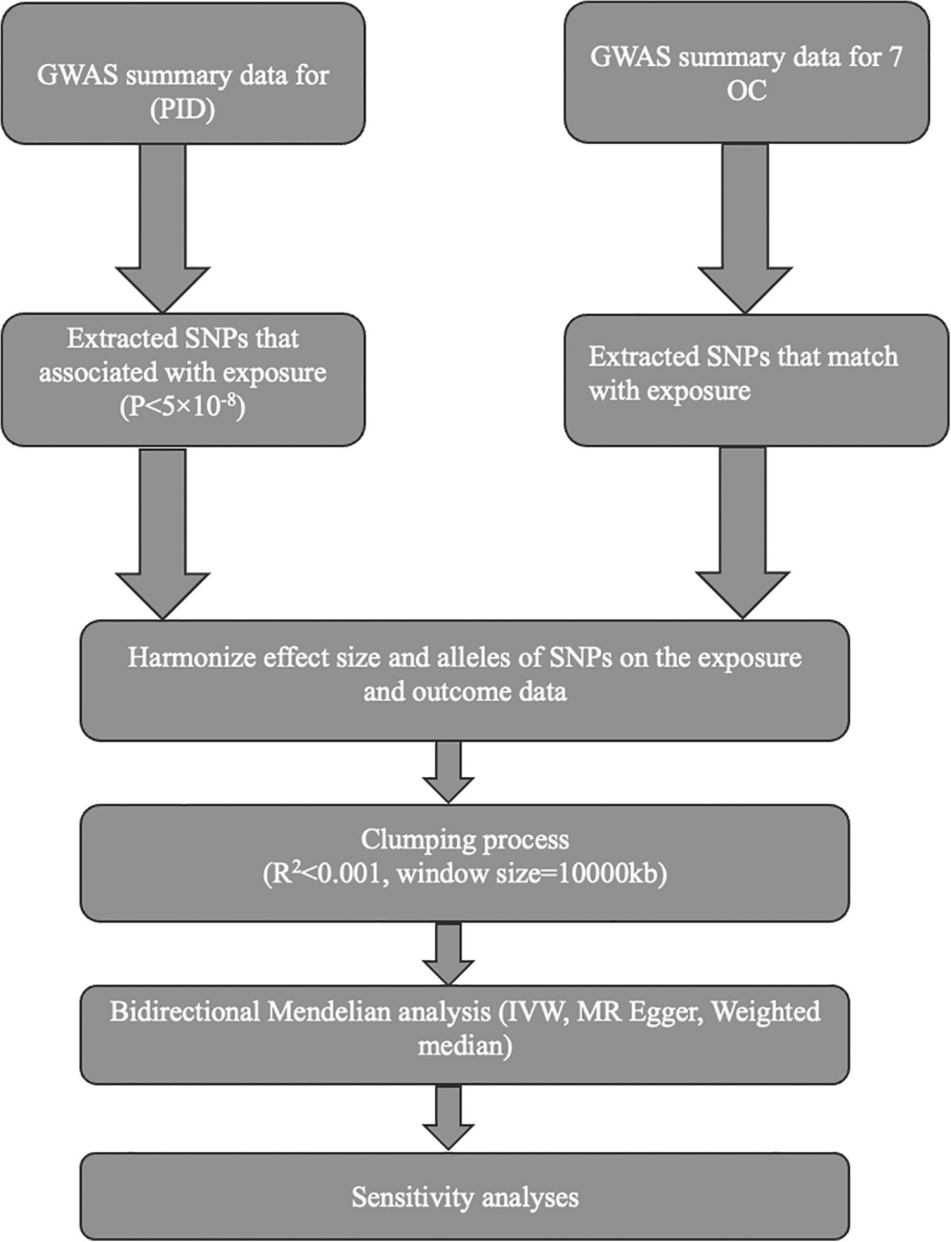
Specific screening procedures for SNPs. GWAS = genome-wide association study, IVW = inverse-variance weighting, MR = Mendelian randomization, OC = ovarian cancer, PID = pelvic inflammatory disease, SNPs = single nucleotide polymorphisms.

SNPs concerning PID with the phenocode “finn-b-N14_OTHFEMPELINF” were chosen as IVs supplied by the genome-wide association study (GWAS) dataset of FinnGen (https://www.finngen.fi/en), and a GWAS was conducted on PID (*F* = 18). The GWAS indicated the PID outcome (including 5681 cases and 205,362 controls). All participants were Europeans. We extracted SNPs based on their genome-wide levels of significance (*P* < 5 × 10^−8^; Fig. 1).

We acquired data for 7 types of OC from the IEU OpenGWAS project (https://gwas.mrcieu.ac.uk). The sample sizes for each were as follows: OC (25,509 cases/40,941 controls), MOC (2566 cases/40,941 controls), LMPOC (3103 cases/40,941 controls), LGSOC (1012 cases/40,941 controls), HGSOC (13,073 cases/40,941 controls), EndoOC (2810 cases/40,941 controls), and CCOC (1366 cases/40,941 controls). Details of these GWAS studies are provided in Table 1.

**Table 1:** Details of these GWAS studies.

Trait	Study	Consortium	Cases/controls	GWAS ID	*F*-statistic
Pelvic inflammatory diseases	2021	Other female pelvic inflammatory diseases	5681/205,362	finn-b-N14_OTHFEMPELINF	18
OC	Phelan 2017^[22]^	Ovarian Cancer Association Consortium	25,509/40,941	ieu-a-1120	30
MOC			2566/40,941	ieu-a-1231	22
LMPOC			3103/40,941	ieu-a-1233	25
LGSOC			1012/40,941	ieu-a-1122	NA
HGSOC			13,037/40,941	ieu-a-1121	28
EndoOC			2810/40,941	ieu-a-1125	NA
CCOC			1366/40,941	ieu-a-1124	NA

*F* = (*R*^2^/[1 − *R*^2^]) × ([N − *k* − 1]/*k*). NA: because no SNPs were available for the reverse analysis.

CCOC = clear cell ovarian cancer, EndoOC = endometrioid ovarian cancer, GWAS = genome-wide association study, HGSOC = high-grade serous ovarian cancer, LGSOC = low-grade serous ovarian cancer, LMPOC = low malignant potential ovarian cancer, MOC = mucinous ovarian cancer, OC = ovarian cancer, SNPs = single nucleotide polymorphisms.

### 2.2. Selection of genetic IVs

To ensure the validity of genetic variants as IVs in MR, the selected SNPs must satisfy 3 core assumptions: SNPs must be strongly associated with the exposure; SNPs should be independent of confounders; and SNPs should influence the outcome solely through the exposure, with no horizontal pleiotropy (HP). We followed standard procedures for SNP selection based on the exposure GWAS summary statistics.

First, SNPs were selected based on genome-wide significance (*P* < 5 × 10^−8^) and were pruned to avoid linkage disequilibrium by using an *R*^2^ threshold of <0.001 within a 10,000-kb window^[23]^, to prevent bias due to correlated variants. Second, exposure SNPs were extracted from the outcome GWAS, and SNPs associated with the outcome (*P* < 5 × 10^−8^) were excluded to maintain the exclusion restriction assumption. SNPs with incompatible alleles or palindromes were removed to ensure allele alignment.^[24]^

Third, MR pleiotropy residual sum and outlier (MR-PRESSO) global tests were performed to identify and remove potential outliers driven by HP. Finally, we calculated *R*^2^ and *F*-statistics for each SNP. SNPs with an *F*-statistic ≥ 10 were retained, as this threshold indicates sufficient instrument strength. SNPs with an *F*-statistic < 10 were excluded to avoid weak instrument bias.^[25]^

To assess potential sample overlap, we examined the exposure and outcome datasets. The FinnGen and OC GWAS datasets were derived from independent consortia, with no substantial sample overlap, thus minimizing the risk of bias from overlapping samples.

### 2.3. Statistical analysis

Three MR methods (inverse-variance weighting [IVW], MR-Egger regression, and weighted median) were adopted to calculate causality. IVW results were used as the primary outcome.^[26]^ In our research, a stochastic effects model was applied to IVW because it maintained conservative estimations even when heterogeneity was present. The MR-Egger regression model offered correspondingly robust estimations independent of IV validity. Nevertheless, MR-Egger methods are susceptible to outliers, leading to relative imprecision and low efficacy.^[27]^ When at least half of the IVs were effective, the weighted median approach examined the median effect across the total instrumental SNPs, with a view to producing an unbiased estimation of the effect.^[28]^ The *P* value for the Bonferroni correction was set at 0.0071 (0.05/7 endpoints), while *P* < .05 was considered nominally significant.

Sensitivity analyses were performed to assess potential heterogeneity and HP in the MR estimates. Directional HP was evaluated using the MR-Egger intercept test, with a *P* value ≥ .05 indicating no statistically significant evidence of directional pleiotropy. Heterogeneity among the genetic variants was assessed using Cochran’s *Q* test, with a *P* value ≥ .05 indicating no statistically significant evidence of heterogeneity. In addition, for the purpose of testing the consistency of each SNP, a leave-one-out (LOO) test was implemented, eliminating each genetic variant one after the other. On condition that causality remains statistically significant after excluding nonspecific SNPs, this provides more plausible evidence for the association. All statistical tests were performed using the “TwoSampleMR” package^[29]^ (version 0.5.7; MRC Integrative Epidemiology Unit, University of Bristol) and the “MR-PRESSO” package^[30]^ in the R software (version 4.2.3; R Foundation for Statistical Computing).

## 3. Results

### 3.1. Forward MR analysis: causal effect of PID on OC

**Figure 2.**
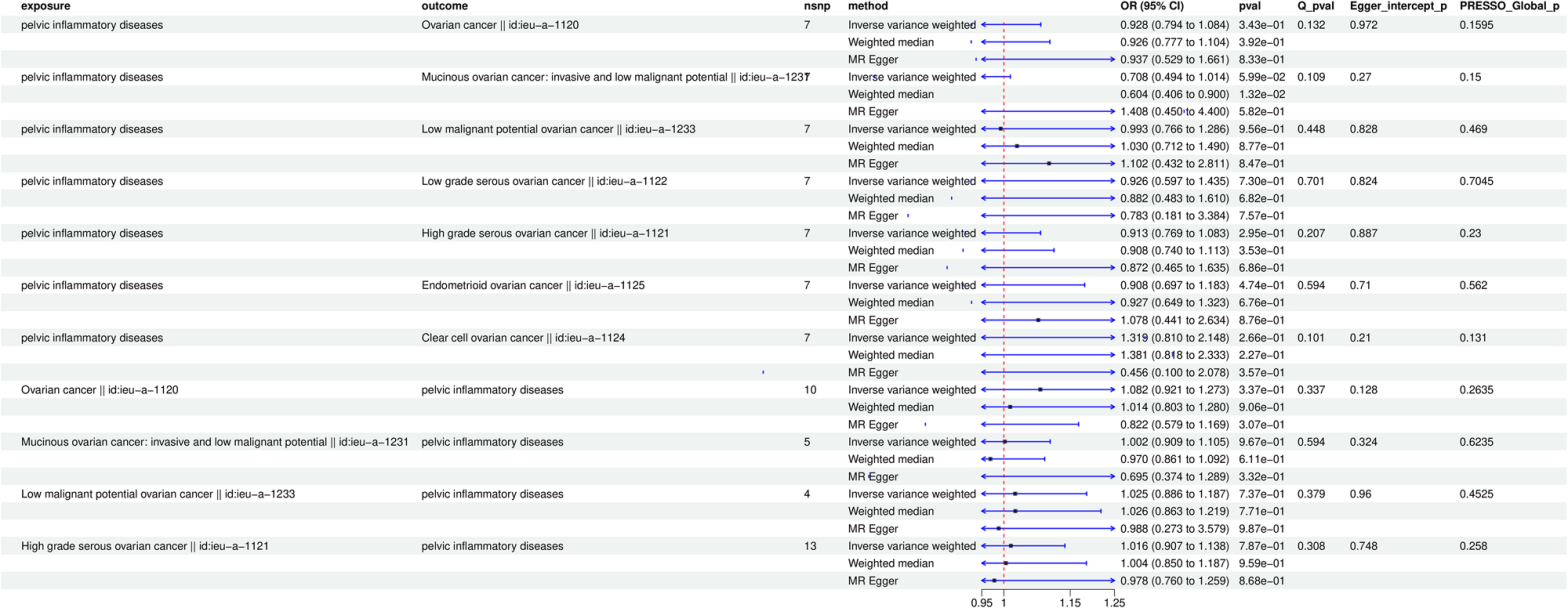
Association between PID and OC. OC = ovarian cancer, PID = pelvic inflammatory disease.

Setting PID as the exposure, we included 7 independent SNPs with significant *P* values <5 × 10^−8^, and our two-sample Mendelian Randomization analysis showed that there was no significant causal relationship between PID and OC, as shown in Figure 2. The results of the IVW analysis showed that PID incidence risk was not significantly associated with OC (odds ratio [OR] = 0.928, 95% CI: 0.794–1.084, *P* = 3.34e−01), MOC (OR = 0.708, 95% CI: 0.494–1.014, *P* = 5.99e−02), LMPOC (OR = 0.993, 95% CI: 0.766–1.286, *P* = 9.56e−01), LGSOC (OR = 0.926, 95% CI: 0.597–1.435, *P* = 7.36e−01), HGSOC (OR = 0.913, 95% CI: 0.769–1.083, *P* = 2.95e−01), EndoOC (OR = 0.908, 95% CI: 0.697–1.183, *P* = 4.74e−01), or CCOC (OR = 1.319, 95% CI: 0.810–2.148, *P* = 2.66e−01). The MR-Egger, weighted median, MR-PRESSO, and weighted mode methods also produced reliable results. There was no heterogeneity among the SNPs. The results of the MR-Egger regression and MR-PRESSO global tests indicated that HP was unlikely to distort the etiology of OC in PID (Fig. 2). LOO analyses showed that no SNP was responsible for the etiological estimates of PID and OC. LOO analysis plots are shown in Supplementary Figures S1, S2, S3 S4 S5 S6 and S7, Supplemental Digital Content 1.

### 3.2. Reverse MR analysis: causal effect of OC on PID

When we performed the reverse MR analysis, LGSOC, EndoOC, and CCOC did not have enough SNPs for analysis. Setting OC as the exposure, we included independent SNPs with *P* values less than 5 × 10^−8^. As shown in Figure 2, our two-sample Mendelian Randomization analyses indicated that there did not appear to be a significant causal relationship between OC and PID. IVW analyses showed that OC (OR = 1.082, 95% CI: 0.921–1.273, *P* = 3.37e−01), MOC (OR = 1.002, 95% CI: 0.909–1.105, *P* = 9.67e−02), LMPOC (OR = 1.025, 95% CI: 0.886–1.187, *P* = 7.37e−01), and HGSOC (OR = 1.016, 95% CI: 0.907–1.138, *P* = 7.87e−01) were not significantly associated with PID. Reliable results were also obtained by the MR-Egger method, the weighted median method, the MR-PRESSO method, and the weighted mode method. There was no heterogeneity among the SNPs based on the heterogeneity test. The results of the MR-Egger regression and the MR-PRESSO global test indicated that HP was unlikely to distort the etiology of PID in OC (Fig. 2). LOO analyses showed that no SNP was responsible for the etiological estimates of PID and OC. Supplementary Figures S8, S9, S10 and S11, Supplemental Digital Content 2, show the LOO analysis plots.

The lack of significant associations across the 7 OC subtypes may stem from multiple factors. First, the genetic variants (SNPs) associated with PID as an exposure variable may exhibit weak links to OC risk, failing to adequately capture the underlying genetic mechanisms between PID and OC. Second, subtype-specific etiology, tumor biological differences, and environmental factors may also contribute to these nonsignificant results. Thus, these findings suggest a complex causal relationship between PID and OC, potentially requiring larger, more diverse samples and optimized genetic tools in future studies.

Regarding the discrepancy between the weighted median and MR-Egger estimates versus the IVW estimates, the primary cause lies in the differing methodological assumptions. The IVW method assumes no pleiotropy and utilizes all SNPs, whereas the MR-Egger and weighted median methods account for pleiotropy and may be more sensitive to bias in certain SNPs. Although these differences are not statistically significant, all methods consistently indicate no significant causal effect between PID and OC, thereby enhancing the robustness of the results.

## 4. Discussion

As far as we know, this study presents the first large-scale two-sample MR analysis aimed at investigating potential causal relationships between PID and 7 types of OC. Unlike conventional observational research, MR analysis helps mitigate confounding factors, offering a clearer picture of the causal relationship between diseases from a genetic perspective. Our bidirectional MR analysis found no causal relationship between PID and OC risk. The null result in this study does not rule out the possibility that other biological mechanisms, such as chronic inflammation, hormonal influences, or infection-mediated pathways, could still be involved in ovarian carcinogenesis, particularly in specific populations or contexts. This highlights that while our study did not find a causal link, PID could still play a role in OC through indirect mechanisms not captured by MR.

Inflammation has been recognized as a well-established promoter of the development of several cancer types since the 19th century^[31]^ and has also been implicated in ovarian carcinogenesis.^[32,33]^ Inflammation involves the release of free radicals, growth factors, cytokines, and adrenergic hormones that have the potential for genetic and epigenetic changes in DNA, including mutations in tumor suppressor genes, which increase the risk of tumor transformation.^[34]^ In addition, cell death associated with inflammation leads to a compensatory increase in cell proliferation, thereby increasing the risk of DNA replication errors. Consistent with our findings, 2 case-control studies^[35,36]^ and a pooled analysis of 13 case-control studies^[37]^ also found no association between PID and OC risk. While our MR study did not identify a genetic link between PID and OC, the complex interplay between inflammatory processes and cancer biology warrants further exploration, especially in the context of specific histologic subtypes of OC. In contrast, results from 1 case-control study^[38]^ and 2 cohort studies^[14,39]^ suggest that women with a history of PID are at increased risk of OC, with a relative risk ranging from 1.9 to 4.0. These findings may be influenced by confounding factors, including socioeconomic status, reproductive history, or lifestyle factors, that could not be fully controlled in these studies. Additionally, the observational design of these studies, which can be affected by reverse causality, further complicates the interpretation of the results. However, OC is a highly heterogeneous disease, with variations in risk factors, potential mutations, and origins among histologic subtypes,^[40]^ and analyses across all OC subtypes may miss histologic subtype-specific correlations; however, only a few studies have examined the role of PID in ovarian carcinogenesis based on histologic subtype.^[13,41]^

We considered the following possible reasons for the discrepancy between the results of the clinical observational study and our MR study. First, there are unavoidable confounders in the data collected in the clinical observational study, which affect the outcomes. Second, the randomized sample of the clinical observational study may have been different from that of our MR study, with many studies choosing to include multiethnic and multiracial samples in the analyses to expand the sample size, while our study only included a sample of European origin. Third, the results of the clinical observational study represent only a possible association between exposure and outcome rather than a clear causal association.

The present study has several strengths. First, the data in this study are derived from the GWAS summary statistics, which were obtained from the largest and most recent studies. In addition, there are no overlapping samples in this study. This greatly improves the statistical power of causal inference. Second, the main advantage of the MR design is that confounders and reverse causality are avoided. Third, all included SNPs had an *F*-statistic of 10 or more, and thus the genetic instruments included were relatively powerful. Fourth, 3 complementary MR analyses were used with multiple sensitivity analyses to reduce the rate of false positives, thus ensuring the precision of our conclusions.

However, there are still some limitations to this study. First, while it is difficult to determine the extent of specimen overlap, there may be some overlap between the exposure and outcome study participants. Fortunately, the methodology used in this study was excellent, with an *F*-statistic significantly >10, which is needed to minimize any potential bias due to specimen overlap. Second, issues such as RNA editing, DNA methylation, and transposon inactivation are inevitable drawbacks of magnetic resonance analyses. Third, there may still be overlap between the genetic variants used in MR and environmental or lifestyle factors that could influence the outcomes, leading to potential residual confounding. Fourth, we did not take into account gender when analyzing the data, as there were no data available on the subject, and whether gender differences would affect the results of the analysis remains to be further investigated. However, given the biological plausibility and the use of a multistage statistical approach, a strict multiple-test correction may be overly cautious and may lead to oversight of the causal relationship between PID and OCs. Furthermore, when interpreting the results of MR analyses, it is crucial to ensure consistency with other measures, such as instrumental strength and sensitivity analyses conducted in our study. Therefore, we remain optimistic about the academic significance of this study. Future studies should aim to validate our current findings with larger sample sizes and different populations to establish the robustness of the results we obtained. It would also be worthwhile to repeat the analyses using different magnetic resonance methods to confirm the identified genetic relationships. Additionally, repeating the analyses with alternative MR methods could help validate the robustness of our findings and shed light on the potential mechanisms underlying the observed associations.

## 5. Conclusion

In this study, the causalities between PID and 7 OCs were comprehensively assessed, but the identified genetic evidence suggested no possible causalities between PID and 7 OCs in European populations.

## Acknowledgments

This work benefited from the publicly available statistics of GWAS. We thank the contributors to the original GWAS database.

## Author contributions

**Conceptualization:** Kaiyun Qin, Yan Jiang, Xingshuang Gao, Weilan Liu, Wenfei Wu, Junmei Zhang, Yuan Zhang, Zhaoping Chu.

**Data curation:** Kaiyun Qin, Yan Jiang, Weilan Liu, Wenfei Wu, Junmei Zhang, Zhaoping Chu.

**Formal analysis:** Kaiyun Qin, Yan Jiang, Xingshuang Gao, Weilan Liu, Yuan Zhang.

**Software:** Yan Jiang, Xingshuang Gao, Weilan Liu, Wenfei Wu, Junmei Zhang, Yuan Zhang.

**Funding acquisition:** Xingshuang Gao, Junmei Zhang, Yuan Zhang, Zhaoping Chu.

**Resources:** Xingshuang Gao, Junmei Zhang.

**Investigation:** Kaiyun Qin, Weilan Liu, Wenfei Wu, Junmei Zhang, Zhaoping Chu.

**Methodology:** Kaiyun Qin, Xingshuang Gao, Wenfei Wu, Junmei Zhang, Yuan Zhang, Zhaoping Chu.

**Project administration:** Kaiyun Qin, Yan Jiang, Xingshuang Gao, Weilan Liu, Wenfei Wu, Zhaoping Chu.

**Supervision:** Kaiyun Qin, Xingshuang Gao, Weilan Liu, Wenfei Wu, Junmei Zhang, Zhaoping Chu.

**Validation:** Kaiyun Qin, Yan Jiang, Weilan Liu, Wenfei Wu, Junmei Zhang, Yuan Zhang, Zhaoping Chu.

**Visualization:** Kaiyun Qin, Yan Jiang, Xingshuang Gao, Weilan Liu, Junmei Zhang, Zhaoping Chu.

**Writing – original draft:** Kaiyun Qin, Yan Jiang, Xingshuang Gao, Weilan Liu, Wenfei Wu, Junmei Zhang, Yuan Zhang, Zhaoping Chu.

**Writing – review & editing:** Zhaoping Chu.






















