# Supplementary figures and images for: Association between pelvic inflammatory disease and ovarian cancer: A bidirectional Mendelian randomization study
Source: Medicine (Baltimore). 2026 Jul 24;105(30):e49820. doi: 10.1097/MD.0000000000049820 (PMC13406073; doi:10.1097/MD.0000000000049820)

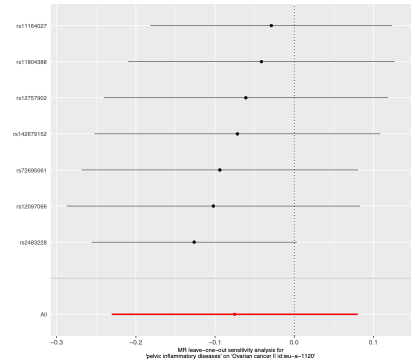

Supplement: Supplementary file 1 [file medi-105-e49820-s001.tiff]

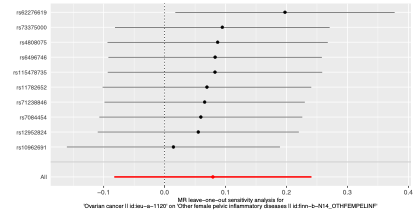

Supplement: Supplementary file 2 [file medi-105-e49820-s002.tiff]

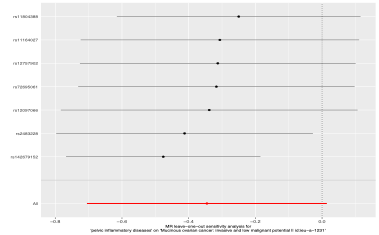

Supplement: Supplementary file 3 [file medi-105-e49820-s003.tiff]

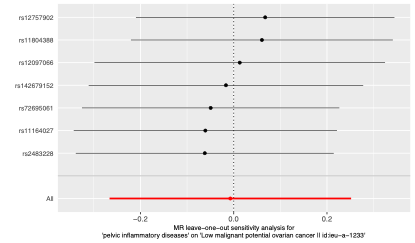

Supplement: Supplementary file 4 [file medi-105-e49820-s004.tiff]

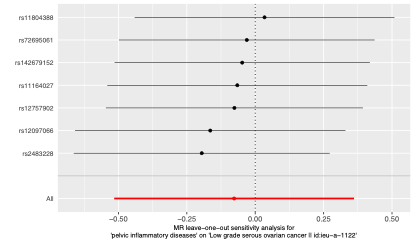

Supplement: Supplementary file 5 [file medi-105-e49820-s005.tiff]

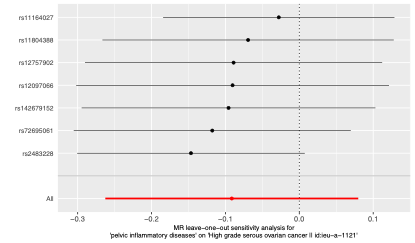

Supplement: Supplementary file 6 [file medi-105-e49820-s006.tiff]

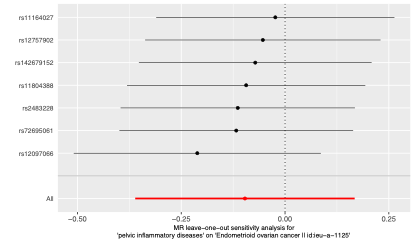

Supplement: Supplementary file 7 [file medi-105-e49820-s007.tiff]

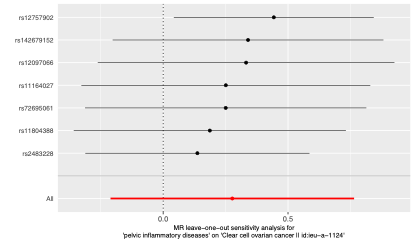

Supplement: Supplementary file 8 [file medi-105-e49820-s008.tiff]

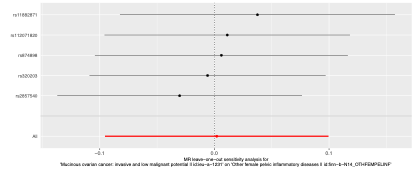

Supplement: Supplementary file 9 [file medi-105-e49820-s009.tiff]

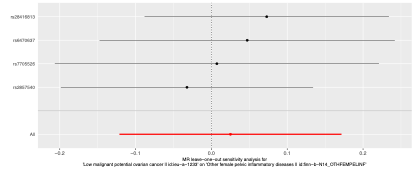

Supplement: Supplementary file 10 [file medi-105-e49820-s010.tiff]

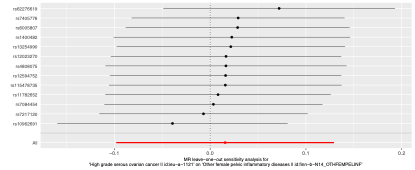

Supplement: Supplementary file 11 [file medi-105-e49820-s011.tiff]
